# Lifestyle changes among people with type 2 diabetes are associated with participation in online groups and time since diagnosis

**DOI:** 10.1186/s12913-021-06660-5

**Published:** 2021-07-12

**Authors:** Anne Helen Hansen, Silje C. Wangberg, Eirik Årsand

**Affiliations:** 1 University Hospital of North Norway, UiT The Arctic University of Norway, PO Box 35, 9038 Tromsø, Norway; 2 Department of Community Medicine, Faculty of Health Sciences, University Hospital of North Norway, PO Box 35, 9038 Tromsø, Norway; 3 Department of Health and Caring Sciences, UiT The Arctic University of Norway, Tromsø, Norway; 4 Norwegian Centre for E-health Research, University Hospital of North Norway, Tromsø, Norway; 5 Department of Computer Science, UiT The Arctic University of Norway, Tromsø, Norway

**Keywords:** eHealth, Internet, Lifestyle, Online support groups, Cross-sectional study, Type 2 diabetes, Norway

## Abstract

**Background:**

For people with Type 2 diabetes (T2D), lifestyle changes may be the most effective intervention. Online groups for people with diabetes holds a great potential to support such changes. However, little is known about the association between participation in online groups and lifestyle changes based on internet information in people with T2D. The aim of this study was to investigate the association between self-reported lifestyle changes and participation in online groups in people with T2D.

**Methods:**

We used e-mail survey data from 1,250 members of The Norwegian Diabetes Association, collected in 2018. Eligible for analyses were the 540 respondents who reported to have T2D. By logistic regressions we studied the association between self-reported lifestyle changes and participation in online groups. Analyses were adjusted for gender, age, education, and time since diagnosis.

**Results:**

We found that 41.9 % of the participants reported lifestyle changes based on information from the internet. Only 6 % had participated in online groups during the previous year. Among those with a disease duration of less than 10 years, 56.0 % reported lifestyle changes, whereas 33.4 % with a disease duration of 10 years or more did so. The odds for lifestyle changes were more than doubled for those who participated in online groups. People who had been diagnosed with diabetes for less than 10 years were significantly more likely to change their lifestyle compared to those with a longer disease duration.

**Conclusions:**

Lifestyle changes based on information from the internet among people with T2D are associated with participation in online groups. Lifestyle changes are also associated with time since diagnosis, making the first years after a T2D diagnosis particularly important for lifestyle interventions. People with T2D, web site developers, online group moderators, health care services, and patient organisations should be aware of this important window for lifestyle change, and encourage participation in online groups.

**Supplementary Information:**

The online version contains supplementary material available at 10.1186/s12913-021-06660-5.

## Background

Type 2 diabetes (T2D) is partly caused, maintained, and deteriorated by preventable risk factors such as physical inactivity, unhealthy diet, obesity, and smoking. For many patients, lifestyle changes may be the most effective intervention [1]. Furthermore, lifestyle changes after a T2D diagnosis reduce the risk for cardiovascular and other adverse events [2]. However, lifestyle changes are often demanding and require information as well as various kinds of support, including planning and self-monitoring tools, feedback, rewards, and social support. Most of these are readily available online. In this study we investigate how lifestyle changes based on information from the internet are associated with participation in online groups in people with T2D.

### Diabetes on the rise

The prevalence of diabetes is on the rise worldwide, expected to affect more than 640 million people in 2040 (age group 20–79 years) [3]. Global prevalence in adults is estimated at 8.8 % [3] and the Norwegian prevalence at 4.7 % [4]. Around 245,000 persons are diagnosed with diabetes in Norway, of whom around 217,000 have T2D [4]. These are most often diagnosed in late adulthood; however, many are undiagnosed.

### eHealth and lifestyle

World Health Organization defines eHealth as “the use of information and communication technologies (ICT) for health” [5]. The use of eHealth is increasing [6]. Today, almost all Norwegian households (98 %) have Internet access, 90 % use the internet every day, 92 % use the internet for e-mail, and 85 % use social media [7–10]. Among people with T2D in Norway, 78 % use eHealth sometimes or often [6, 11]. In 2013, 61 % in a general Norwegian population had read about exercise or diet on the internet, 17 % had posted a status about exercise or diet on a social network site (SNS), and 18 % had kept an online exercise or diet journal [12]. One in ten had discussed exercise or diet online with peers, and 7 % had used electronic communication to ask professionals [12].

### Online support groups

A consistent research finding is that eHealth used with additional support or face-to face-interventions increase the effectiveness compared to stand-alone eHealth interventions [13–15]. Online support groups (OSGs) for people with diabetes have shown to be valuable support in disease management [16]. These groups are run without involvement of health personnel. Today, Facebook is the platform for most and largest OSGs in Norway. There are groups for people with any diabetes type, typically with around 10,000 users. In addition, there are more specific groups discussing technical equipment (insulin pumps, glucose sensors etc.), with between 500 and 2,000 participants, mostly people with type 1 diabetes but increasingly also people with T2D. Groups have been established by committed people who are typically not satisfied with the support they get from health care services, need someone to discuss disease related issues with, and recently also by people who want to join their forces in making alternative and better technical solutions than the commercial actors and health care services offer today. Most OSGs are closed, with group moderators who are partly responsible for the content and for accepting new members.

Persons with diabetes affiliated with a clinic in USA reported the following reasons for visiting a diabetes SNS: to offer or receive support or encouragement, to share personal experiences, or to give or seek advice about clinical diabetes care and obtain advice about lifestyle [17]. Both giving and receiving advice about lifestyle was positively correlated with adherence to healthy diet, days with more than 30 min of physical activity, and with more constructive discussions with health personnel regarding diabetes care. In another American study, older people with diabetes reported that the internet offer a source of information and support that they were unable to find elsewhere [18]. Other studies confirm the psycho-social effects of information exchange and social support online, whereas evidence on physical effects are somewhat limited [19].

### Relevance of the study

The use of eHealth varies between countries, regions, diagnostic groups, health care services, and health care systems. Hence, research on the impact of OSGs in different settings is important to achieve a comprehensive epidemiological understanding of its influence. Research on how lifestyle changes relates to participation in online groups among people with T2D is largely lacking. This is of particular interest for patients and patient organizations, health care providers, administrators, and policy makers. Increased knowledge will make us better able to plan for future eHealth interventions, aiming for a healthy lifestyle with prevention of disease and disease complications at the lowest possible cost.

### Aim

The aim of this study was to investigate the association between self-reported lifestyle changes based on information from the internet and participation in online groups in people with T2D in Norway. Based on international research and our experiences, we hypothesized a positive association in this regard.

## Methods

### Study design and setting

For this cross-sectional study we used questionnaire data obtained by e-mail from members of The Norwegian Diabetes Association (NDA). By 31.12.2017, the organization had 33,908 members, 53 % women and 47 % men. Around 70 % were diagnosed with T2D [20]. The Norwegian Centre for Research Data (NSD) Web Survey distributed the invitations to a randomly selected sample of 5,971 individuals (about 18 % of all members).

We developed a comprehensive questionnaire (Additional file 1), using relevant questions from other published surveys on information seeking and lifestyle aspects [21, 22]. This included questions about use of the internet for health purposes, experiences with eHealth, self-reported lifestyle changes from online health information, participation in online groups, duration and management of diabetes, as well as demographic and socio-economic information.

Before data collection (January and February 2018), the questionnaire was reviewed and tested by persons diagnosed with diabetes and experts from our research group.

Information about the study purpose and what participation would entail was distributed together with the invitation. One reminder was submitted to non-respondents by e-mail 15 days after the first request. The participants did not receive any form of compensation for participating.

### Participants

**Fig. 1:**
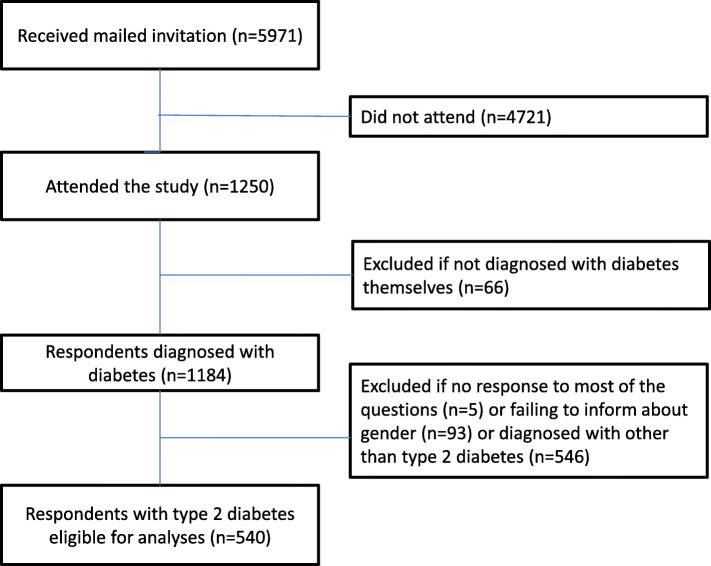
Flow chart of study population

 It was not possible for the participants to respond more than once. From the 1,250 participants, we excluded those who did not suffer from diabetes themselves (n = 66). This group consisted of 61 family members, 4 health personnel (2 overlapping), and 3 others. Further, we excluded those who failed to respond to most of the questions (n = 5), those who did not inform about gender (n = 93), and those who reported other diabetes types than type 2 (n = 546). The sample finally consisted of 540 respondents (Fig. 1).

### Variables

The variable self-reported lifestyle changes was obtained from the question “Based on the information you have found on the internet, have you changed your lifestyle?” The original answering options were never, once, sometimes and often. Since we were interested in whether the participants had ever made any lifestyle changes, we dichotomized this variable into never (no) and once/sometimes/often (yes).

The variable participation in online groups was derived from the question “During the past 12 months, have you taken part in any online group for people with diabetes?» Responses were coded as yes/no.

We grouped age in 20-year intervals. The four education categories were labeled low (primary/part of secondary school), middle (completed secondary school), high (college/university < 4 years), and highest (college/university 4 years or more). Diabetes duration was grouped in 10-year intervals, however, the participants with 30 years or more since diagnosis were merged into one group.

The response time variable was constructed as a dichotomous variable consisting of early and late respondents in the two groups.

### Analyses

Data were analyzed by means of descriptive statistics and logistic regressions. Correlations were tested with Spearman’s as well as Pearson’s correlation coefficients. The dependent variable in the multivariable regression model was self-reported lifestyle changes. The independent variables were introduced collectively into the model.

Due to a relatively low response rate, we compared respondents who did not respond initially but eventually consented with early respondents, assuming that late respondents were more similar to non-respondents [23]. This was done by subsequently introducing the response time variable into the regression model.

We used 95 % confidence intervals (CI) and set *p* < 0.05 throughout the study. All analyses were accomplished using Stata, version 16.1. All methods were performed in accordance with relevant guidelines and regulations.

## Results

### Participation

Altogether 1250 persons years participated, giving an estimated participation rate of 21 % (1250/5971). Since the registered e-mail addresses might not have been completely updated, and since we received more than 400 bounce backs from servers unable to deliver the invitation, the true response rate is assumed to be higher. After exclusions described in the methods section (Fig. 1), 540 individuals with T2D were eligible for analyses.

### Characteristics of the participants

Mean age of participants was 62.4 years, 60.0 years for women and 63.8 years for men. The youngest participant was 22 years, and the oldest 89 years. Median age was 63 years. Mean disease duration was 12.7 years, whereas median disease duration was 10 years. Mean age at diagnosis was 49.6 years, whereas median age was 51 years.

The largest groups were made up of men, people aged 60 years and over, people with middle education, and a disease duration of less than 10 years (Table 1).

**Table 1:** Sample characteristics

	Total sample type 2 diabetes	
	**n**	**%**
**Gender (*n* = 540)**		
Female Male	202 338	37.4 62.6
**Age (*n* = 540)**		
18–39 40–59 60 years and over	14 171 355	2.6 31.7 65.7
**Education**^**a**^**(*n* = 506)**		
Low Middle High Highest	68 163 148 127	13.4 32.2 29.3 25.1
**Diabetes duration (*n* = 537)**		
< 10 years 10–19 years 20–29 years 30 years and over	206 201 100 30	13.4 32.2 29.3 25.1
**Lifestyle changes (*n* = 513)**		
Never Once Sometimes Often	298 35 151 29	58.1 6.8 29.4 5.7
**Participation in online groups (*n* = 519)**		
No Yes	488 31	94.0 6.0

^a^: Low (primary/part of secondary school), Middle (high school), High (college/university < 4 years), Highest (college/university 4 years or more)

Nearly half of the participants reported that they had made lifestyle changes based on information from the internet (215/513, 41.9 %). Among these, most participants answered “sometimes” rather than “once” or “often” (Table 1). Only 6 % (31/488) had participated in online groups during the previous year (Table 1).

**Fig. 2:**
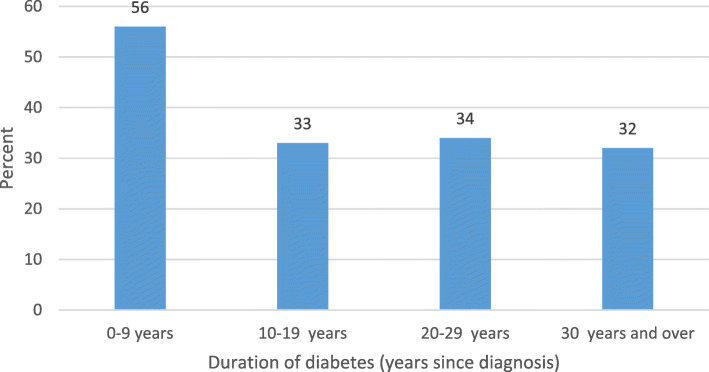
Self-reported lifestyle changes by duration of diabetes

Among those who had been diagnosed with T2D for less than 10 years, 108/193 (56.0 %) reported lifestyle changes (Fig. 2). Among those with a disease duration of 10 years or more, 107/320 (33.4 %) reported lifestyle changes.

### Associations of lifestyle changes

The odds for lifestyle changes were significantly higher for those who had participated in online groups during the previous year (odds ratio [OR] 2.56, confidence interval [CI] 1.13–5.83). Likewise, the odds for lifestyle changes was significantly higher in the group with highest education (OR 2.13, CI 1.10–4.10), compared to the low education group.

People who had been diagnosed with diabetes for 10 years or more were significantly less likely to change their lifestyle, as the odds for changing lifestyle were more than halved compared to those with a disease duration of less than 10 years (Table 2).

**Table 2:** Probability of lifestyle changes in people with T2D (*N* = 496)

	OR	p	CI
**Participation in online groups**			
No^a^ Yes	1.00 **2.56**	**0.024**	**1.13–5.83**
**Gender**			
Female^a^ Male	1.00 0.85	0.433	0.58–1.27
**Age**			
18–39 40–59 60 years and over	1.00 0.66 0.39	0.556 0.194	0.16–2.67 0.10–1.61
**Education**^**b**^			
Low^a^ Middle High Highest	1.00 1.69 1.72 **2.13**	0.102 0.095 **0.024**	0.90–3.17 0.91–3.26 **1.10–4.10**
**Diabetes duration**			
< 10 years^a^ 10–19 years 20–29 years 30 years and over	1.00 **0.45** **0.50** 0.52	**< 0.001** **0.014** 0.143	**0.29–0.70** **0.29–0.87** 0.21–1.25

Statistically significant findings are marked in bold

*OR* odds ratio; *CI* confidence interval

^a^: Reference groups

^b^: Low (primary/part of secondary school), Middle (high school), High (college/university < 4 years), Highest (college/university 4 years or more)

Gender and age were not associated with lifestyle changes (Table 2).

Introduction of the response time variable into the regression model did not alter the results significantly.

There were no strong correlations (defined as Spearman’s rho > 0.5) between the independent variables. A similar result was found using Pearson’s correlation test.

## Discussion

### Key findings

Nearly half of the participants reported that they had made lifestyle changes based on information from the internet. Only 6 % had participated in online groups during the previous year. Among those with a disease duration of less than 10 years, 56.0 % reported lifestyle changes, whereas only 33.4 % with a disease duration of 10 years or more did so. The odds for lifestyle changes were more than doubled for those who had participated in online groups. Likewise, the odds for lifestyle changes were significantly higher among the highest educated, compared to the lowest educated. People who had been diagnosed with diabetes for more than 10 years were significantly less likely to change their lifestyle compared to those more recently diagnosed.

### Lifestyle changes based on internet information

Lifestyle changes based on information from the internet were reported by 41.9 % of the participants. We do not know what kind of lifestyle changes these were, nor to what degree changes were made and whether they sustained. The percentage of reported lifestyle changes was low in our study compared to a study of seniors in the Netherlands (mean age 72 years), where 56 % reported lifestyle changes as a result of information from the internet (2011). A study from USA found that 69 % of respondents with diabetes were moderately to extremely likely to follow advice they received from a website about lifestyle changes for diabetes [17]. These surveys are small, and needs to be replicated and extended, however, the findings show the great potential of internet-based information in supporting lifestyle changes. This is underlined by a systematic evaluation of 224 relevant reports on internet and mobile interventions among generally healthy adults, concluding that internet interventions improved diet, physical activity, adiposity, smoking, and excess alcohol, whereas mobile interventions improved physical activity and adiposity [13].

### Participation in online groups

Only 6 % of the participants reported that they had participated in online groups during the previous year. In a USA study, 31 % reported using diabetes specific social media sites [17]. However, percentages are not directly comparable due to content and wording of questions, study setting, and gender and age of study participants. Study participants were younger in the clinic-based American study, most were women, and social media users were significantly younger than non-users, which add to explaining at least part of the differences [17]. In our study, participants reported lifestyle changes without limiting it to the previous year, whereas participation in online groups were reported for the previous year. We anticipate that participation in OSGs during the previous year might partly be an extension of participation in earlier years, and that active participation might vary according to perceived needs at different times. OSGs seem to hold a great potential for increased participation among people with T2D in Norway.

### Positive association between lifestyle changes and participation in online groups

We found that the odds for lifestyle changes were more than doubled for those who participated in online groups compared to those who did not. Direct comparable studies are lacking, but the finding is not surprising, and in line with research confirming that one of the users’ reasons for visiting social network sites is to get lifestyle advice [17]. We know that diet plans, recipes, and eating tips are widely discussed in OSGs [16]. One of the reasons that participation in online groups is associated with lifestyle changes, might be the group’s contribution in assessing whether the internet information is of high quality, and reliable. Our result suggests that people with T2D should be encouraged, for instance by patient organisations and health care personnel, to participate in OSGs.

### Association between lifestyle changes and time since diagnosis

Our finding that people with less than 10 years since T2D diagnosis are more likely to report lifestyle changes is not surprising and in concordance with common sense and general beliefs.

We could not find solid evidence in previous eHealth research directly comparable to this result. However, an Australian study without eHealth focus investigated lifestyle behaviours according to duration of newly diagnosed T2D, finding that no positive lifestyle changes were associated with increased time since diagnoses, even if magnitude of weight changes and walking increased with time [24]. Changes in moderate to vigorous physical activity, number of cigarettes smoked, and smoking cessation decreased with time since diagnosis [24]. Receiving a diagnosis of diabetes or other chronic disease including an encouragement of lifestyle changes might be a motivator, however, motivation might fade with time [25, 26].

Our finding is particularly interesting, since it suggests a window for change that patients, patient organisations, health care personnel, and web site developers should be aware of. From these findings, people with T2D might benefit from a special focus on lifestyle changes during the first years after diagnosis. Lifestyle focus might for instance take the form of information seeking on the internet and personal invitations to join OSGs. Internet and OSGs are available all day, and night, and can provide information and support when people are ready for it. Available resources on an ongoing basis are useful and required, especially for patients who experience a delay between their registered needs and desires, and a more structured and planned education after receiving a diagnosis.

### Association between lifestyle changes and education

We found no associations of lifestyle changes with gender and age. On the other hand, we found that the highest education group had higher odds for lifestyle changes based on internet information, compared to the lowest education group. An educational divide is consistent in most previous research on education and online health information seeking, in general as well as in disease specific populations [27–29]. However, in recent research this has been nuanced as an educational divide among people with T2D has been confirmed for the use of search engines, but not for the use of social media [11], both being a prerequisite for lifestyle changes in this study. In general, it is well known that women, younger people and the higher educated have a lifestyle closer to recommended health behaviours. These groups also have the lowest prevalence of T2D. Summing up these lines, it is not surprising that the highest educated are more likely to make lifestyle changes when diagnosed with T2D.

### Limitations

Even if the true response rate is assumed to be higher than estimated, the low participation rate is a limitation of this study. However, our analyses regarding a possible recruitment skewness in this regard, comparing early respondents with late respondents, revealed no significant differences between the groups.

The distribution of the questionnaire by e-mail excluded participants without an e-mail address. However, since most Norwegians use e-mail, we do not think this has affected our results significantly. Nevertheless, some might not use e-mail regularly, and some may use mobile phones more than computers, both involving barriers to filling out a questionnaire. Also, people with interest in eHealth might be overrepresented, as interest in the topic studied might increase responses [30]. In this case, our rates of lifestyle changes and participation in online groups might be higher than the true rates. However, these aspects will apply to most eHealth research.

Our questions regarding lifestyle changes were not specified regarding type and amount of changes. The reported lifestyle changes may range from diet and physical activity to smoking cessation, however, the changes have been regarded large and important enough to be reported by each of the participants.

We also recognize that the low participation in online groups is a study limitation. However, this is adjusted for by the p-values and the confidence intervals.

In questionnaire data there is always a potential for recall bias, particularly regarding minor events and distant past. In addition, the validity of self-reported data may be questioned. Overreporting of lifestyle changes is likely to a certain degree, since there is always a risk in surveys that participants will appear better than reality [31].

Our study might also be limited by a possibly greater engagement in treatment and lifestyle changes among members of NDA, and in particular those participating in OSGs, compared to others diagnosed with T2D. If this is the case, our results might be more positive than what is generalizable for the general T2D population. However, there are evidence that engagement in health protective behaviour may be similar across the population, whether diagnosed with diabetes or not [32].

In sum, it is not possible to judge the magnitude of any possible biases, since different factors might pull the tendency in different directions, or level each other out. However, our results seem reasonable in an area lacking solid and updated investigations. We suggest that bias poses a limited threat to our study’s validity, nevertheless, generalization must be made with caution.

### Strengths

This study focuses on an area that has been scarcely investigated, recruiting adult participants from all of Norway. The collection of data in cooperation with the Norwegian Diabetes Association enabled us to develop excellent user participation throughout the project with a large and important group of eHealth and health care users.

## Conclusions

This is one of few studies mapping the relation between lifestyle changes based on information from the internet and participation in online groups. We conclude that there is a positive association between lifestyle changes based on information from the internet among people with T2D and participation in online groups. Lifestyle changes are also associated with time since diagnosis, making the first years after a T2D diagnosis particularly important for lifestyle interventions. People with T2D, web site developers, online group moderators, health care services, and patient organisations should be aware of this important window for lifestyle change, and encourage participation in online groups. Internet and online groups hold an important potential to provide information and support when people are ready for it, and in the way they prefer. Further research is required to highlight the present findings in larger samples with chronic disease, and in general populations, all in need for a preventive and health-promoting lifestyle.

## Supplementary Information

**Additional file 1: **Questionnaire.

## Data Availability

The data that support the findings of this study are available from the Norwegian Centre for Research Data (NSD) but restrictions apply to the availability of these data, which were used under license for the current study, and so are not publicly available. Data are however available from the authors upon reasonable request and written permission of NSD and the Data Protection Officer (Personvernombudet) at the University Hospital of North Norway.

## Abbreviations

NDA: Norwegian Diabetes Association
NSD: The Norwegian Centre for Research Data
REK: Regional Committee for Medical and Health Research Ethics
OR: Odds ratio
CI: Confidence interval
T2D: Type 2 diabetes
